# SULF2 Expression Is a Potential Diagnostic and Prognostic Marker in Lung Cancer

**DOI:** 10.1371/journal.pone.0148911

**Published:** 2016-02-16

**Authors:** Natalie S. Lui, Yi-Wei Yang, Annemieke van Zante, Petra Buchanan, David M. Jablons, Hassan Lemjabbar-Alaoui

**Affiliations:** 1 Thoracic Oncology Program, Department of Surgery, University of California San Francisco, San Francisco, California, United States of America; 2 Department of Pathology, University of California San Francisco, San Francisco, California, United States of America; Istituto dei tumori Fondazione Pascale, ITALY

## Abstract

**Aims:**

Lung cancer is one of the most deadly cancers; median survival from diagnosis is less than one year in those with advanced disease. Novel lung cancer biomarkers are desperately needed. In this study, we evaluated SULF2 expression by immunohistochemistry and its association with overall survival in a cohort of patients with non-small cell lung cancer (NSCLC). We also looked for the presence of SULF2 protein in plasma to evaluate its potential as an early detection biomarker for NSCLC.

**Methods:**

We identified patients who underwent surgical resection for pulmonary adenocarcinoma or squamous cell carcinoma at our institution. A section from each paraffin-embedded specimen was stained with a SULF2 antibody. A pathologist determined the percentage and intensity of tumor cell staining. Survival analysis was performed using a multivariate Cox proportional hazards model. Using a novel SULF2 ELISA assay, we analyzed plasma levels of SULF2 in a small cohort of healthy donors and patients with early stage NSCLC.

**Results:**

SULF2 staining was present in 82% of the lung cancer samples. Squamous cell carcinomas had a higher mean percentage of staining than adenocarcinomas (100% vs. 60%; p<0.0005). After adjusting for age, sex, race, histologic type, stage, and neoadjuvant therapy, there was a non-significant (31%; p = 0.65) increase in the risk of death for patients with adenocarcinoma with SULF2 staining in tumor cells. In contrast, there was a significant decrease in the risk of death (89%; p = 0.02) for patients with squamous cell carcinoma with SULF2 staining in tumor cells. SULF2 protein was present in plasma of patients with early stage NSCLC, and soluble SULF2 levels increased with age. Finally, plasma SULF2 levels were significantly elevated in early stage NSCLC patients, compared to healthy controls.

**Conclusions:**

Tumor expression of SULF2 may affect prognosis in NSCLC, while blood SULF2 levels may have a significant role in the diagnosis of this fatal disease.

## Introduction

Lung cancer is the most frequently diagnosed non-cutaneous malignancy and the leading cause of cancer death worldwide [1–4]. There are two major histologic categories of pulmonary carcinoma: small cell lung carcinoma (SCLC) and non-small cell lung carcinoma (NSCLC). NSCLC accounts for about 90% of all lung cancers and is comprised of three subcategories: adenocarcinoma (ADC), squamous cell carcinoma (SCC), and large-cell carcinoma [5]. The majority of lung cancers are attributable to cigarette smoking, which exposes the airways to tobacco smoke carcinogens. Active smoking increases the risk of developing lung cancer by 13-fold and long-term passive exposure to cigarette smoke by 1.5 fold [2]. Approximately 90% of all lung cancer-related deaths are caused by tobacco use [6]. As with other cancers [7], there is no single mutation that is responsible for lung cancer, but a succession of molecular changes contribute to tumor formation. Lung cancer is a heterogeneous disease involving somatic mutations and epigenetic dysregulation of a number of signaling pathways. Several Receptor Tyrosine Kinases (RTKs) (EGFR, IGF-1R, and cMet), as well as the GTPase Kras, have been implicated as oncogenes, whereas loss-of-function mutations are most frequently found in p53, RB and p16INK4a [1,2]. Despite the improvements in diagnosis and therapy made during the past 25 years, the prognosis for patients with lung cancer is still poor [8,9]. Further understanding of the molecular basis of lung cancer, including the discovery of disease-specific biomarkers, would greatly improve our ability to diagnose, provide prognostic information, and potentially guide treatment of patients.

Heparan sulfate proteoglycans (HSPGs) consist of core proteins that are modified by the covalent addition of HS chains containing variably sulfated repeating disaccharide units [10]. HSPGs carry out innumerable signaling functions, using their sulfated chains to bind diverse protein ligands, such as growth factors, morphogens, chemokines, and cytokines. These ligand interactions largely depend on the pattern and density of the sulfation modifications with 6-O-sulfation of glucosamine (6OS) known to be key [11]. Two recently discovered sulfatases (SULF1 and SULF2) provide a novel mechanism for the regulation of HSPG-dependent signaling by removing 6OS. SULFs are neutral pH, extracellular enzymes which remove 6OS from intact heparin/HSPGs; they promote key signaling pathways by mobilizing protein ligands (e.g., Wnt, GDNF, PDGF-B, BMP-4) from HSPG sequestration, thus liberating the ligands for binding to signal transduction receptors [12].

One or both SULF transcripts are broadly overexpressed in many human cancers, including NSCLC, glioblastoma, hepatocellular carcinoma, breast cancer, head and neck cancer, pancreatic adenocarcinoma, multiple myeloma, and gastric carcinoma [11,13]. SULF2 has been directly implicated as a driver of carcinogenesis in NSCLC [14], murine and human malignant glioma including glioblastoma and oligodendroglioma [15,16], pancreatic cancer [17], and hepatocellular carcinoma [18]. The most thorough investigation of the role of SULF2 in cancer has been carried out by us in NSCLC. Briefly, in NSCLC we found [14]: 1) upregulation of both SULFs at the transcript level; 2) SULF2 protein expression in 20 of 20 human NSCLC tumors with minimal levels in normal lung; 3) SULF2 protein promotes the in vitro malignant phenotype, and the tumorigenicity in mice of SULF-2 positive human NSCLC cell lines; and 4) SULF2 promotes human lung carcinogenesis by regulation of Wnt signaling and the kinase activity of three critical receptors (i.e., EGFR, IGF-1R and cMet) (unpublished). Dysregulation of each of these three receptors has been causally linked to lung cancer development, progression, and increased resistance to chemotherapy [19–21]. These findings demonstrate that SULF2 regulates a number of signaling pathways that are subject to aberrant activation in cancer. This regulation is exerted upstream of the interaction of growth factors with RTKs and the activation of intracellular kinases. Moreover, SULF2 promoter methylation and expression has been associated with overall survival in lung cancer, gastric cancer, and hepatocellular carcinoma [6,7]. The objective of the present study was to evaluate SULF2 expression by immunohistochemistry and correlate this with disease progression and overall survival in a cohort of patients with NSCLC.

## Methods

### Patients

We identified 121 patients who underwent lung cancer resection for either invasive ADC (52 patients) or invasive SCC (69 patients) at our institution from 2000 to 2006. We excluded patients with recurrent lung cancer, no available paraffin-embedded tissue specimen, or less than 3 mm of invasive tumor on an H&E slide. Clinicopathologic data was obtained through review of electronic medical records. Histologic data was obtained through review of pathology reports and confirmed by review of H&E sections by a pathologist (AvZ). Pathologic stage was determined by the American Joint Committee on Cancer (AJCC) staging system, 7^th^ edition [8]. Vital status was obtained through the Social Security Death Master File. Overall survival was calculated from the date of surgery. The UCSF institutional review board approved this study. Patients’ records and information were anonymized and de-identified prior to analysis.

### Immunohistochemistry

A 5-μm section from each paraffin-embedded tissue specimen was stained with a mouse monoclonal antibody to SULF2 (2B4) [12] at a concentration of 2μg/ml with avidin-biotin blocking. This antibody was previously developed and validated [12]. A pathologist (AvZ) blinded to patient outcome determined the percentage and intensity of tumor cell staining. The percentage of tumor cell staining was scored from 0 to 100%. The intensity of tumor cell staining was assessed at 100x magnification and scored from 0 to 3. A score of 0 represented no staining; 1, weak staining; 2, moderate staining; and 3, strong staining. When specimens showed a range of intensity, the mean intensity was recorded. The presence of endothelial cell staining was assessed for each slide and functioned as an internal positive control. Staining of tumor-associated stroma was also noted.

### SULF2 Sandwich ELISA

Sandwich ELISA for detection of SULF2 in blood was performed as previously described [22]. Briefly, for quantification of SULF2 in plasma from NSCLC patients, the assay was performed with 12.5% diluted plasma. Triplicate determinations were conducted for each plasma sample. Background values were determined with mIgG1 substituting for anti-SULF2 antibody as the capture reagent. The mean background value was subtracted from that with the anti-SULF2 capture antibody to yield the specific signal, which was used to compute SULF2 by comparison with the SULF2 standard run on the same plate. Plasma samples that had backgrounds greater than twice the intrinsic background (mIgG1 capture signal with no serum added) or yielded a negative value were excluded.

### Statistical Analysis

Patient baseline characteristics and immunohistochemistry scores were summarized and compared by histologic type, using the Student's t-test for continuous variables and the chi-squared test for categorical variables. Survival analysis was performed using univariate and multivariate Cox proportional hazards models. Age, sex, and race were included in the multivariate model a priori. Histologic type, stage, grade, neoadjuvant therapy, and year of operation were included in the multivariate analysis only if the p-value was less than 0.10 in the univariate analysis. We repeated our analyses in pre-specified subgroups by histologic type (ADC and SCC) and neoadjuvant therapy (yes and no). For all statistical tests, a two-sided alpha level of 0.05 was considered statistically significant. Analyses were performed using Stata version 11.

## Results

### Patients

**Fig 1:**
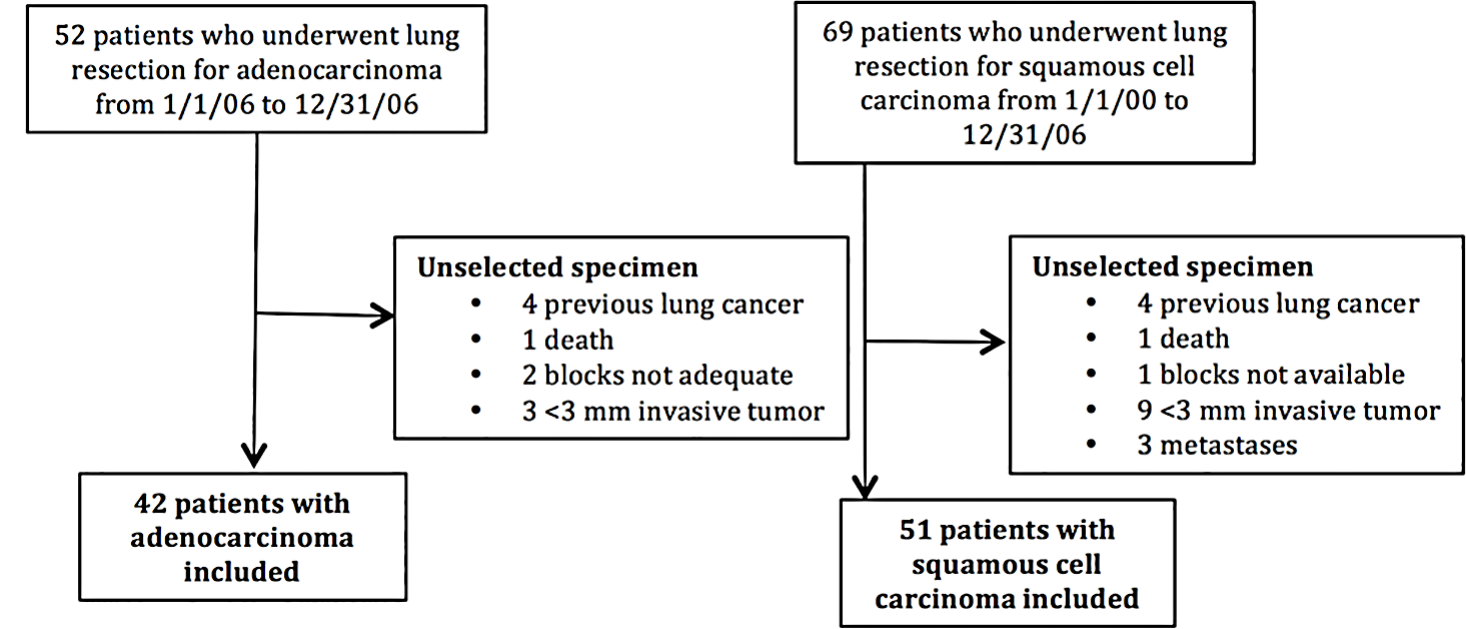
Patient selection. Patient selection by review of 121 patients who underwent lung resection for lung cancer during the 6-year period from 2000 to 2006.

We identified 121 patients who underwent surgical resection for lung cancer at our institution during the 6-year period from 2000 to 2006. Ninety-three of these 121 patients met our inclusion criteria, including 42 patients with ADC and 51 patients with SCC (Fig 1).

There were several differences in baseline characteristics between patients with ADC and those with SCC (Table 1). Patients with SCC had a higher proportion of men (69% vs. 43%; p = 0.012), were more likely to be past or current smokers (90% vs. 76%; p = 0.006), were more likely to undergo pneumonectomy (14% vs. 2%; p = 0.03), and had a higher frequency of neoadjuvant therapy (47% vs. 26%; p = 0.039).

**Table 1:** Baseline characteristics by histologic type of 93 patients underwent lung cancer resection for adenocarcinoma (ADC) (41 patients), or lung squamous cell carcinoma (SCC) (51 patients) at UCSF from 2000 to 2006. *p Values were calculated using the t test for continuous variables and the x^2^ test for categorical variables.

	All Patients (N = 93)	Patients with ADC (N = 42)	Patients with SCC (N = 51)	p Value*
Age, mean ± SD—yrs.	68.1 ± 10.1	66.5 ± 11.2	69.3 ± 9.1	0.19
Sex—no. (%)				0.012
Female	40 (43)	24 (57)	16 (31)	
Male	53 (57)	18 (43)	35 (69)	
Race—no. (%)				0.003
White	74 (80)	28 (67)	46 (90)	
Asian	11 (12)	10 (24)	1 (2)	
Black	3 (3)	2 (5)	1 (2)	
Missing	5 (5)	2 (5)	3 (6)	
Hispanic—no. (%)				0.08
No	84 (90)	35 (83)	49 (96)	
Yes	2 (2)	1 (2)	1 (2)	
Missing	7 (8)	6 (14)	1 (2)	
Smoking status—no. (%)				0.006
Never	12 (13)	10 (24)	2 (4)	
Past or current	78 (84)	32 (76)	46 (90)	
Missing	3 (3)	0	3 (6)	
Neoadjuvant therapy—no. (%)				0.039
No	58 (62)	31 (74)	27 (33)	
Yes	35 (38)	11 (26)	24 (47)	
Resection—no. (%)				0.03
Wedge resection	16 (17)	9 (21)	7 (14)	
Lobectomy	69 (74)	32 (76)	37 (73)	
Pneumonectomy	8 (9)	1 (2)	7 (14)	
Incision—no. (%)				0.042
VATS	16 (17)	12 (29)	4 (8)	
Thoracotomy	75 (81)	30 (71)	45 (88)	
Other	2 (2)	0	2 (4)	
Stage—no. (%)				0.356
I	56 (60)	29 (69)	27 (53)	
II	18 (19)	5 (12)	13 (25)	
III	14 (15)	6 (14)	8 (16)	
IV	4 (4)	2 (5)	2 (4)	
Grade—no. (%)				<0.0005
1	17 (18)	15 (36)	2 (4)	
2	51 (55)	21 (50)	30 (59)	
3	20 (22)	3 (7)	17 (33)	

### Immunohistochemistry

**Fig 2:**
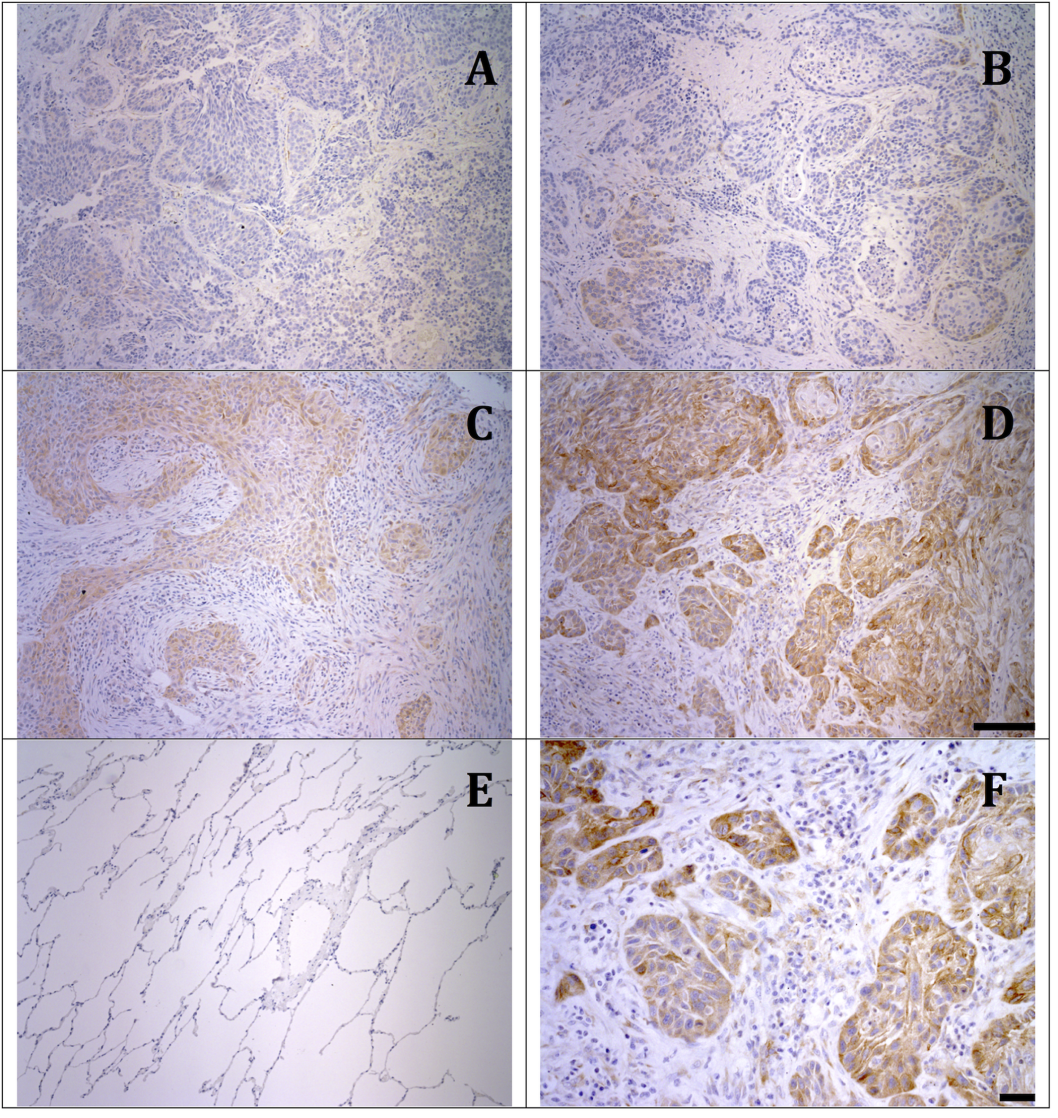
Sulf-2 protein expression in NSCLC tumors. Representative sections from squamous cell carcinoma samples with no staining (A), weak staining (B) and moderate staining (C) strong staining (D), and adjacent normal lung with no staining (E). Panel F shows staining of tumor- associated stromal cells with 2B4 antibody. Panels A, B, C, D, and E are low- power micrographs (10X, scale bar = 100μm). Panel F is a high-power micrographs (20X, scale bar = 50μm).

SULF2 staining in either tumor or stromal cells was detected on 72/93 of the specimens, including 25/42 (60%) of ADC samples and 51/51 (100%) of SCC samples (Table 2). All samples had positive endothelial cell staining, which served as an internal positive control. Strikingly, tumor cell staining was detected in only 19% (8/42) of the ADC cases, as compared to 94% (42/51) of the SCC cases (Fig 2). However, 50% (17/42) ADC and 88% (45/51) SCC samples showed conspicuous staining of tumor stroma including spindle-shaped stromal cells in addition to endothelial cells lining blood vessels. Except for scattered epithelial basal cells, the normal airway epithelium adjacent to tumors was negative for SULF2 staining (Fig 2).

**Table 2:** SULF2 Immunohistochemistry staining results by histologic type of NSCLC tumors. SULF2 staining was positive for 82% of the samples (Either tumor or stroma staining). The squamous cell carcinoma (SCC) samples had a higher mean percentage of tumor staining compared to the adenocarcinoma (ADC) samples (100% vs. 60%; p<0.0005). *p Values were calculated using the t test for continuous variables and the x^2^ test for categorical variables

	All patients (N = 93)	Patients with ADC (N = 42)	Patients with SCC (N = 51)	*p-value
Tumor staining—no. (%)				<0.0005
Yes	56 (60)	8 (19)	48 (94)	
No	37 (40)	34 (81)	3 (6)	
Percent tumor cells staining, mean ± SD	31.4 ± 35.4	8.1 ± 21.7	50.5 ± 32.9	<0.0005
Intensity tumor cells staining, mean ± SD	1.1 ± 1.0	0.3 ± 0.7	1.7 ± 0.8	<0.0005
0	37 (40)	34 (81)	3 (6)	
1	18 (19)	3 (7)	15 (29)	
2	32 (34)	5 (12)	27 (53)	
3	6 (6)	0	6 (12)	
Stroma staining—no. (%)				<0.0005
Yes	66 (71)	21 (50)	45 (88)	
No	26 (28)	21 (50)	6 (12)	
Tumor or stroma staining—no. (%)				<0.0005
Yes	76 (82)	25 (60)	51 (100)	
No	17 (18)	17 (40)	0	

Furthermore, SULF2 staining differed by histological type, likely reflecting their distinct etiologies. SCC samples had a higher mean percentage (50.5% vs. 8.1%; p <0.0005) and intensity (1.7 vs. 0.3; p <0. 0005) of tumor cell staining compared to the ADC samples. However, no correlation was found between either tumor or stromal cell staining and neoadjuvant therapy in either histological type. Additionally, neither percentage nor intensity of tumor or stromal cell staining was significantly associated with the other clinical and pathologic variables, including stage and grade.

### Survival analysis

Median follow-up time was 53.6 months. In the univariate Cox proportional hazards models (Table 3), pathologic stage and age were significantly associated with overall survival. As expected, higher stage was significantly associated with worse overall survival. There was a trend towards an association between overall survival and both neoadjuvant therapy and histologic type. These were included in the multivariate model (Table 4). Interestingly, patients who underwent neoadjuvant therapy had worse overall survival than those who did not. This result likely reflects selection bias in which patients diagnosed with more aggressive tumors are more likely to receive neoadjuvant therapy. Year of surgery and histologic grade were not significantly associated with survival; these were not included in the multivariate model (Table 4). In all patients, presence of SULF2 staining of either tumor cells or stroma was associated with a 42% increase in the risk of death, but this result was not statistically significant (p = 0.39). After adjusting for age, sex, race, histologic type, stage, and neoadjuvant therapy, patients with ADC with SULF2 staining in tumor cells had a 31% increased risk of death (p = 0.65) (Table 4), and patients with SCC with SULF2 staining in tumor cells had an 89% decreased risk of death (p = 0.02) (Table 4).

**Table 3:** Univariate Cox proportional hazard model for overall survival. (*100% of tumors stained positive for either tumor or stroma).

	All patients		Patients with ADC		Patients with SCC	
	HR	p-value	HR	p-value	HR	p-value
Age (per 10 years)	1.45	0.013	1.59	0.058	1.31	0.169
Sex						
Male	1		1		1	
Female	0.78	0.398	0.78	0.617	0.98	0.961
Race						
White	1		1		1	
Asian	1.33	0.491	1.92	0.207	2.34	0.409
Black	-	-	-	-	-	-
Missing	-	-	-	-	-	-
Smoking status						
Never	1		1		1	
Past/Current	1.4	0.48	1.11	0.858	1.31	0.791
Stage						
I	1	0.0017	1	0.09	1	0.0017
II	0.8	0.579	3.5	0.066	0.39	0.066
III	3.46	<0.0005	3.6	0.025	3.8	0.003
IV	1.85	0.312	1.96	0.526	1.65	0.502
Grade						
1	1	0.4945	1	0.959	1	0.6218
2	1.15	0.73	1.14	0.801	0.64	0.547
3	1.61	0.29	0.93	0.95	0.88	0.871
4	-	-	-	-	-	-
Neoadjuvant therapy						
No	1		1		1	
Yes	1.52	0.147	1.95	0.19	1.18	0.636
Histologic type						
Adenocarcinoma	1					
Squamous cell	1.68	0.089				
Tumor cells staining						
No	1		1		1	
Yes	1.58	0.144	1.34	0.611	0.78	0.736
Percent (per 10%)	1.05	0.256	1.1	0.298	0.98	0.712
Intensity						
0	1		1		1	
1	1.44	0.374	2.07	0.34	0.65	0.583
2	1.79	0.084	0.99	0.99	0.95	0.941
3	1	0.998	-	-	0.48	0.427
Stroma cells staining						
No	1				1	
Yes	0.84	0.559	0.65	0.378	0.58	0.232
Tumor or stroma cells staining						
No	1		1			
Yes	1.42	0.393	1.95	0.19	Omit	Omit

**Table 4:** Multivariate Cox proportional hazards models for overall survival (*100% of tumors stained positive for either tumor or stroma).

	All patients		Patients with ADC		Patients with SCC	
	HR	p-value	HR	p-value	HR	p-value
Tumor	0.79	0.69	1.31	0.65	0.11	0.02
Percent (per 10%)	0.99	0.88	1.07	0.48	0.96	0.55
Intensity	0.96	0.85	1.04	0.92	0.88	0.68
Intensity						
0	1		1		1	
1	0.68	0.55	2.89	0.2	0.09	0.01
2	0.82	0.75	0.8	0.78	0.13	0.03
3	0.57	0.53	-	-	0.09	0.03
Stroma	0.56	0.09	0.49	0.16	0.51	0.20
Tumor or Stroma	0.84	0.73	0.68	0.47	Omit*	Omit*
Score	1	0.98	1	0.61	1	0.82

### Analysis of SULF2 in blood

**Fig 3:**
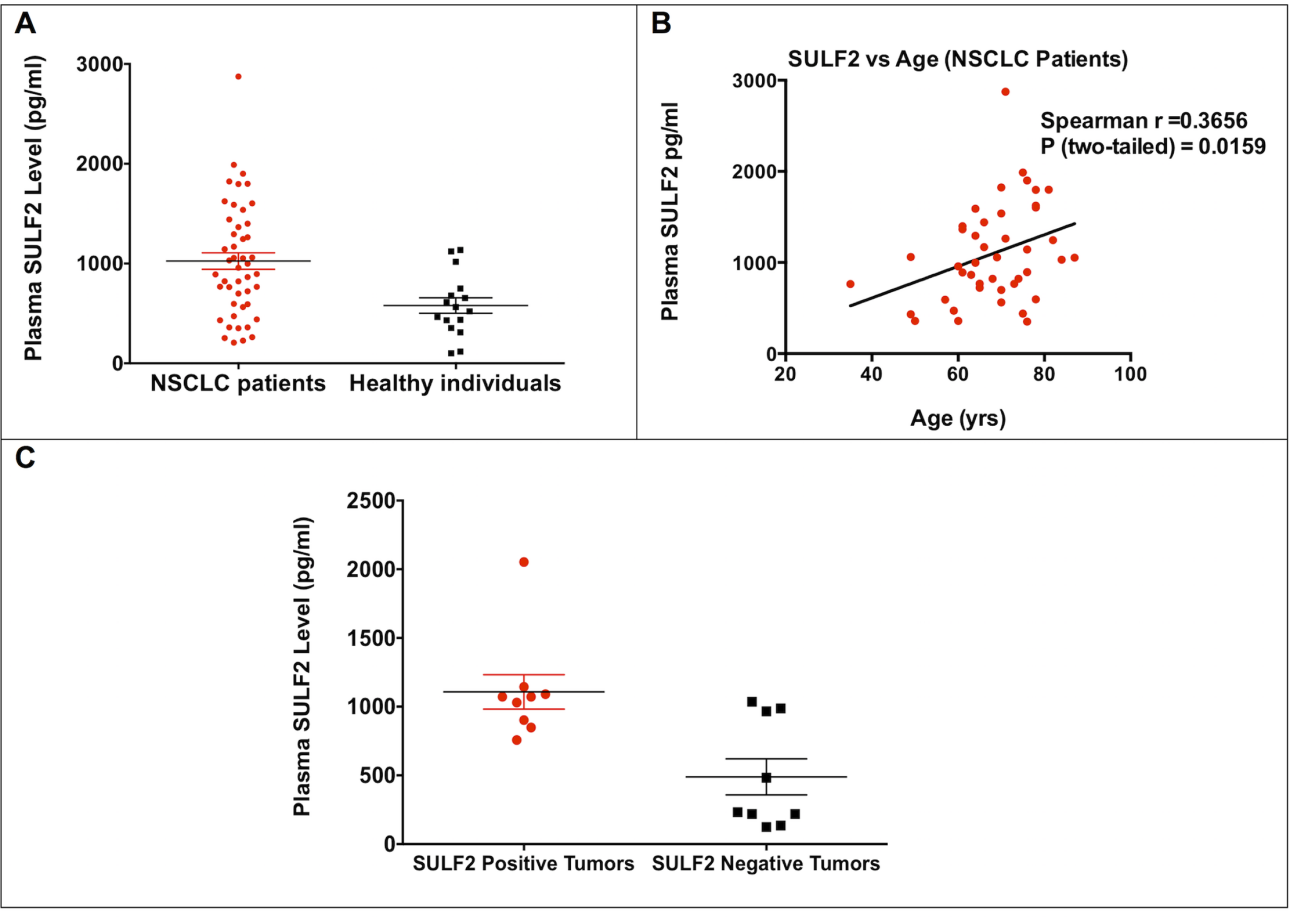
SULF2 protein levels in blood can be quantified in NSCLC patients and healthy individuals. A) Plasma samples from 45 newly diagnosed early stage NSCLC patients and 16 healthy controls were analyzed using SULF2 ELISA assay. SULF2 protein levels in blood are elevated in NSCLC patients ((Mean ± SEM = 1024.2 ± 82.8 pg/ml) relative to healthy individuals controls (Mean ± SEM = 574.1 ± 78.15 pg/ml), (p = 0.004). P value was determined by T-test with welsh correction for unequal variances. B) Significant positive correlation between plasma SULF2 levels and age was found in NSCLC patients (*r* = 0.3656, p = 0159). r and P values were determined by nonparametric two-tailed Spearman correlation. C) SULF2 protein is significantly elevated in plasmas of NSCLC patients with SULF2 positive tumors (Mean ±SEM = 1108.0 ±125.5 pg/ml), as compared with NSCLC patients with SULF2 negative tumors (Mean ±SEM = 489.3 ±131.4 pg/ml) (p = 0.0003). P value was determined by T-test with welsh correction for unequal variances.

Our group previously developed a robust sandwich ELISA to detect SULF2 in human blood (plasma and serum) [22]. For this study we examined plasma SULF2 levels in a cohort of 43 patients with newly diagnosed early stage NSCLC. Plasma was collected prior to surgical resection for lung cancer at our institution. These patients had stage IA or IB (71%) or stage II (29%) NSCLC. Soluble SULF2 was present in the blood of patients with early stage NSCLC (Mean ± SEM = 1024.2 ± 82.8 pg/ml) (Fig 3A). Furthermore, there was an age-associated increase in SULF2 levels in plasma (Fig 3B). This is the first time that SULF2 levels have been quantified in the blood from patients with NSCLC. Importantly, our analyses revealed that SULF2 protein is significantly elevated in plasmas of NSCLC patients with SULF2 positive tumors (Mean ±SEM = 1108.0 ±125.5 pg/ml), as compared with NSCLC patients with SULF2 negative tumors (Mean ±SEM = 489.3 ±131.4 pg/ml) (p = 0.0003) (Fig 3C). Additionally, we analyzed SULF2 levels in commercially available plasmas from 16 healthy donors. Our results revealed that the plasma level of SULF2 protein is markedly increased in NSCLC plasmas, as compared with healthy controls (Mean ± SEM = 1024.2 ± 82.8 pg/ml vs. 574.1 ± 78.15 pg/ml, respectively) (Fig 3A). These data are exciting and provide the first indication that SULF2 blood levels may be useful biomarkers for early detection of lung cancer. However, additional studies are required to validate these findings in blood samples from a bigger cohort of NSCLC patients collected from specified centers with their own inherent healthy control subjects, matched by age, gender and smoking, and collected on the same protocol.

## Discussion

The univariate Cox proportional hazards models revealed that NSCLC patients with a high level of SULF2 expression in tumors as assessed by immunohistochemistry tended to have shorter overall survival, indicating that a high SULF2 level may be a marker of poor prognosis in these patients. Intriguingly, after adjusting for age, sex, race, histologic type, stage, and neoadjuvant therapy, tumor cell staining for SULF2 was associated with a trend towards increased risk of death (31%, NS) in ADC patients. Conversely in patients with SCC, SULF2 staining in tumor cells was associated with a significantly decreased risk of death (89%, p = 0.02). The aforementioned divergence in outcome related to tumor SULF2 staining between ADC and ACC patients, likely reflects differing etiology and tumor behavior of these two lung cancer subtypes. However, It is important to note that the SCC group has a very limited number of SULF2 negative cases (3 out of 49 cases). Hence, our findings need further confirmation in larger cohorts of NSCLC patients to corroborate a possible differential outcome between SCC and ADC associated with SULF2 positive staining in tumors.

As expected, higher stage was also significantly associated with worse overall survival. Interestingly, patients who underwent neoadjuvant therapy had worse overall survival than those who did not. This result likely reflects selection bias, in which patients with more aggressive tumors were more likely to receive neoadjuvant therapy.

This study also showed that SULF2 staining differed by histologic type, likely reflecting their distinct etiologies. Squamous cell carcinoma samples had significantly higher percentage and intensity of tumor cell staining than adenocarcinoma samples. These results are in accord with our earlier findings, in which SULF2 expression in tumor cells by immunohistochemistry was present in ten out of ten lung squamous cell carcinoma samples, but zero out of ten lung adenocarcinoma samples [12]. However, both adenocarcinomas and squamous cell carcinomas showed conspicuous staining of tumor stroma [12].

The association of SULF2 and overall survival corresponds to previous findings in various types of cancer. In our initial investigations, we showed that higher levels of SULF2 protein as assessed by immunochemistry is associated with increased severity of disease and worse survival in patients with esophageal cancer [23]. Lai et al. showed that increased SULF2 transcript expression by microarray was associated with aggressive tumors and poorer survival in patients with hepatocellular carcinoma [24]. Bret et al. demonstrated that SULF2 expression in primary multiple myeloma cells was associated with a poor prognosis in two independent large cohorts of patients [13]. Finally, Tessema et al. and Wang et al. showed that SULF2 promoter methylation was associated with improved overall survival in patients with lung adenocarcinoma and gastric cancer, respectively [25,26]. In keeping, our results show for the first time that SULF2 at the protein level is associated with overall survival in NSCLC, thus confirming its important role in carcinogenesis and potential as a predictive biomarker.

The extracellular endosulfatases (SULF1 and SULF2) are overexpressed in a wide assortment of human cancers [11,13,16] and SULF2, in particular, has been implicated, by us and others, as a driver of carcinogenesis in NSCLC [12], pancreatic carcinoma [17], malignant astrocytoma [15,16], colorectal carcinoma [27], and hepatocellular carcinoma [18]. As extracellular enzymes that are both tethered to the cell membrane and secreted, the SULFs and their Heparan sulfate proteoglycan (HSPG) substrates are present in the extracellular environment. HSPGs carry out innumerable signaling functions by using their sulfated chains to bind diverse protein ligands, such as growth factors, morphogens, and cytokines. These interactions depend on the pattern of the sulfation modifications [10]. The 6-O-sulfation of glucosamine (6OS) is known to be key for many ligand interactions. SULFs selectively remove the critical 6OS modifications from HSPGs, and in so doing, SULFs prevent ligand sequestration, allowing these ligands to interact with their corresponding receptors. This phenomenon has been demonstrated for Wnt, VEGF, FGF-1, PDGF, CXCL12 (SDF-1), CCL21, IL-8, and IP-10 [28,29]. Aberrant activation of the aforementioned signaling pathways has been implicated in many forms of carcinogenesis.

Originally, SULFs were thought to be tumor suppressors [30–32]. This belief originated from experiments in which forced expression of a SULF in several tumor lines caused reduced growth-factor signaling by HB-EGF, FGF-2 or HGF, and diminished tumorigenicity. The negative effects of SULFs on FGF-2 signaling are consistent with the requirement for 6S on HSPGs in the FGF-2 signaling complex [33]. However, consistent with a potential oncogenic role, one or both *SULF* genes were soon found to be overexpressed in subsets of multiple tumors (breast, pancreatic, hepatocellular carcinoma, malignant astrocytoma, head and neck, lung, and multiple myeloma) [11]. In particular, SULF2 has been directly implicated as a candidate cancer-causing gene in human breast cancer and mouse brain cancer [34,35]. Moreover, in malignant astrocytoma, and pancreatic, colorectal, and lung cancer cell lines SULF2 knockdown led to reduced proliferation and decreased growth of xenografts in mice, likely due to its effect on signaling pathways induced by HSPG binding factors [14,16–18,27]. In hepatocellular cancer cell lines, overexpression of SULF2 led to increased proliferation and migration and markedly enhanced the tumorigenicity of these cells in nude mice [18].

Our results show that SULF2 was expressed in 72 of 93 human lung cancer tumors, including 25 of 41 (60%) adenocarcinoma samples and 51 of 51 (100%) squamous cell carcinoma samples. Importantly, high expression of SULF2 was associated with worse prognosis in patients with adenocarcinoma. These findings are in agreement with a previous report showing a significant correlation between SULF2 promoter methylation and survival in lung adenocarcinoma patients [25]. Surprisingly, SULF2 staining of tumor cells in patients with squamous cell carcinoma was associated with a better survival rate. Nonetheless, these findings are very encouraging, and it seems that the majority of adenocarcinomas samples (including early stage disease), and all of the squamous cell carcinoma tumor samples have some degree of staining for SULF2 protein. Together, these data suggests the potential of SULF2 protein as a prognostic biomarker in patients with lung cancer. Moreover, as a secreted molecule that may accumulate in blood or other body fluids, it is also has potential as a diagnostic biomarker.

Both in the USA and worldwide, lung cancer has long been the number-one cause of cancer related death [3,4,36]. Because of its late presentation and diagnosis, lung cancer has a very poor prognosis and poor cure rate [37,38]. The majority of cases (over 60%) are diagnosed at a late stage of the disease where current treatment modalities are unlikely to be effective [35–37]. The 5-year overall survival rate is less than 10% in patients with advanced disease, but greater than 60% in patients with stage 1 disease [3,4]. Therefore, there is a pressing need for effective tools that allow for earlier diagnosis and treatment. There are currently no biomarkers in use clinically due to their lack of adequate sensitivity and specificity. SULF2 is important in this regard as it is a secreted enzyme with a demonstrated relevance to lung carcinogenesis. Hence, the development of SULF2 as a biomarker may help identify a high-risk group that would make screening feasible.

In our present study, 20 out of 34 (59%) early stage (I or II) lung adenocarcinoma samples had SULF2 staining. Also, 40 out of 40 (100%) early stage (I or II) squamous cell lung carcinoma samples had SULF2 staining. As secreted enzymes, we hypothesize that the soluble SULF2 potentially released from tumors may be detected in the blood and thus may serve as a biomarker for the early detection of NSCLC with SULF2 overexpression. To test this hypothesis, we used our recently developed and validated ELISA assay for detection of SULF2 in human blood [22]. We found that plasma from patients with early stage NSCLC contains a mean level of Mean ± SEM = 1024.2 ± 82.8 pg/ml SULF2 (Fig 3A). Additionally, our results suggest that there is an age-associated increase in SULF2 levels in plasma, thus age may be a potential confounding variable. These data provide the first evidence that SULF2 protein can be quantified in blood from a patient with early stage lung cancer. Also, we have performed preliminary analyses of SULF2 levels in blood from early stage NSCLC cancer patients. We found that mean SULF2 levels in blood are significantly elevated in patients with NSCLC and as compared to healthy blood donors (Mean ± SEM = 1024.2 ± 82.8 pg/ml vs. 574.1 ± 78.15 pg/ml, respectively) (Fig 3A). The data suggest that there is an age-associated increase in SULF2 levels in plasma, thus age may be a potential confounding variable. Nevertheless, the results of this initial analysis provide evidence that early stage lung cancer is associated with an increased plasma concentration of SULF2. Therefore, we are providing the first validation of blood SULF2 as a potential biomarker for early detection of cancer using a quantitative ELISA. Moreover, the findings of this initial analysis open the possibility of blood SULF2 as a potential biomarker for early detection of cancer using a quantitative SULF2 ELISA. Although SULF2 detection assay needs further validation on a larger clinical cohort with age- and gender matched controls and may not be suitable as a stand-alone test, its combination with assays that measure the levels of other circulating biomarkers such as CYFRA, may provide sufficient specificity to complement the imaging modalities routinely used in population screening for cancer.

In conclusion, in this study we have shown by immunohistochemistry that SULF2 protein is present in the majority of lung adenocarcinomas and in all of the lung squamous cell carcinoma samples we tested. We showed for the first time that SULF2 staining of tumor cells was associated with a trend towards worse overall survival in patients with lung adenocarcinomas. This is significant as approximately nine of every ten cases of lung cancer can be categorized as NSCLC, and non-squamous NSCLC comprises up to 70% of these cases. Moreover, we have demonstrated that soluble SULF2 is increased in blood from early stage NSCLC patients thus, providing the first evidence of soluble blood SULF2 as a potential biomarker for early detection of NSCLC. Together, these findings are very encouraging and corroborate the potential of SULF2 protein as a diagnostic and a prognostic biomarker in patients with NSCLC.
